# Cerebral Hemodynamic Changes during the Trigeminocardiac Reflex: Description of a New Animal Model Protocol

**DOI:** 10.1100/tsw.2010.136

**Published:** 2010-07-20

**Authors:** N. Sandu, J. Cornelius, A. Filis, C. Nöthen, J. Rasper, V. I. Kulinsky, B. J. Schaller

**Affiliations:** ^1^ Neurosurgical Department, University of Paris, France; ^2^ Neurosurgical Department, University of Erlangen, Germany; ^3^ Neurosurgical Department, University of Münster, Germany; ^4^ Biochemistry Department, Irkutsk State Medical University, Irkutsk, Russia

**Keywords:** trigeminocardiac reflex, trigemino-cardiac reflex, animal model, oxygen-conserving reflex, cerebral hemodynamic, cerebral blood flow

## Abstract

The trigeminocardiac reflex (TCR) is a well-known brainstem reflex, first described in skull base and neurosurgery by the senior author in 1999, leading to reflex apnea, bradycardia, and changes of mean arterial pressure. There seem to be differences between peripheral and central stimulation of the TCR, and there is a lack of clear data about the cerebral hemodynamic changes during the TCR. However, the research of this reflex principally focused on clinical cases for peripheral and central stimulation during the last years, and on rabbits for peripheral stimulation several decades ago, so there was a need for an animal model that allows us to use the current state-of-the-art imaging methods. The new animal model protocol as introduced by the authors gives, for the first time, deep insights into the cerebral hemodynamic changes during the TCR and gives substantial evidence whether the TCR represents an oxygen-conserving reflex or not.

### INTRODUCTION

The trigeminocardiac reflex (TCR) is a brainstem reflex that is defined as the sudden onset of parasympathetic dysrhythmia, sympathetic hypotension, apnea, or gastric hypermotility during stimulation of any of the sensory branches of the trigeminal nerve[1,2,3,4,5,6]. The sensory nerve endings of the trigeminal nerve send neuronal signals via the Gasserian ganglion to the sensory nucleus of the trigeminal nerve, forming the afferent pathway of the reflex arc[3,7]. This afferent pathway continues along the short internuncial nerve fibers in the reticular formation to connect with the efferent pathway in the motor nucleus of the vagus nerve[3,7]. Kumada et al. ablated the nucleus tractus solitarius (NTS) and found that the TCR was not diminished, although fibers of the trigeminal nerve have been seen to join the NTS[1,2]. In addition, it was observed through localized stimulation that the most sensitive sites for the

TCR were in the dorsal spinal tract[1,2]. Others have shown connections between the trigeminal ganglion and the vagus[8,9]. Lang et al. described short internuncial fibers originating from the trigeminal sensory nucleus under the floor of the fourth ventricle and ending in the efferent pathway of the reticular formation, which originates from the motor nucleus of the vagus[9]. Increased tone of the efferent vagal fibers leads to the autonomic changes of the TCR[10,11,12,13].

The TCR was experimentally first described by White et al. in 1969[14] and later as an entity by Kumada et al. in 1977 as a nasopharyngeal reflex through their neurostimulation experiments on rabbits[1,2]. Clinically, the TCR has been reported to occur during different skull base procedures[3,4,5,6,10,15,16,17,18,19,20,21,22,23,24,25,26,27,28,29,30,31,32,33]. Generally accepted, we were the first to elicit the TCR by central stimulation in clinical neurosurgery in 1999[3]. Apart from the few, but increasing, clinical reports that underline the TCR's effect on outcome[3,4,5,6,7,8,25,26,29,32,33,34,35,36), the detailed physiological function of this brainstem reflex has not yet been fully explored. Recently, we suggested that all these different reflex forms (e.g., nasopharyngeal reflex, oculocardiac reflex) should be subsumed under the term "TCR", but with differing into peripheral or central reflex stimulation[4,31,33]. From experimental findings in rabbits, it may be suggested that the TCR represents an expression of a central neurogenic reflex leading to rapid cerebrovascular vasodilatation generated from excitation of oxygen-sensitive neurons in the rostral ventrolateral medulla oblongata[4]. However, these data have so far only been investigated in the cortex[37]. By this physiological response, the adjustments of the systemic and cerebral circulations are initiated to divert blood to the brain or to increase blood flow within it[7].

In addition to different clinical observations of the TCR[3,4,5,6,10,13,14,15,16,17,18,19,20,21,22,23,24], there is only one experimental report among the experimental studies[35,37,38,39,40,41] that deals with changes in the cerebral blood flow (CBF) after the TCR[37], but these data are only representative for the cortex. No data exist that document the influence of the TCR on the CBF in the short- and long-term follow-up period after initiation of the reflex. Some important results can be oxygen-conserving reflexes and ischemic tolerance. We present here a new animal model for the TCR. With diffusion-weighted imaging (DWI) and perfusion-weighted imaging (PWI), used here, the *in vivo* (ischemic) changes associated with altered CBF have been widely investigated[42,43].

### THEORETICAL BACKGROUND

The treatment of hemodynamic instability after occurrence of the TCR consists of ceasing manipulation of the tissue. Then, light anesthesia, hypoxia, hypercapnia, and acidosis should be corrected because all can potentiate the TCR. Often, the arrhythmias weaken with repetitive stimulation. If the dysrhythmia continues or recurs despite these maneuvers, anticholinergic drugs should be administered and immediate cardiac life support initiated as needed. Atropine or glycopyrrolate have both been shown to extinguish the TCR effectively. Anticholinergic drugs are not given prophylactically because they can cause refractory arrhythmias[44,45]. The use of fentanyl to block pain receptors does not prohibit the TCR. It has been demonstrated repeatedly that the use of an anticholinergic is sufficient to blunt the TCR and that the reflex weakens with repeated stimulation, but if these techniques fail, it is possible to pace the heart externally if necessary[3].

#### The Hemodynamic Changes

The brain can protect itself from ischemia by two main mechanisms that probably involve two separate systems of neurons in the central nervous system. One, which mediates a reflexive neurogenic neuroprotection, emanates from oxygen-sensitive sympathoexcitatory reticulospinal neurons of the rostroventrolateral medulla[4]. These cells, excited within seconds by reduction in blood flow or oxygen, initiate the systemic vascular components of the oxygen-conserving (diving) reflex[46]. They profoundly increase regional CBF without changing regional cerebral glucose utilization and, hence, rapidly and efficiently provide the brain with oxygen[38]. After cessation of the stimulus, the systemic and cerebrovascular adjustments return to normal. The system mediating reflex protection works via as-yet-undefined projections from the rostroventrolateral medulla to the upper brainstem and/or thalamus to engage a small population of neurons in the cortex that appear to be dedicated to transduce a neuronal signal into vasodilation[38]. It also appears to relay the central neurogenic vasodilation elicited from other brain regions, including excitation of axons. This mode of protection would be initiated under conditions of global ischemia and/or hypoxemia because the signal is detected by medullary neurons. The second neuroprotective system is represented in intrinsic neurons of the cerebellum and mediates a conditioned central neurogenic neuroprotection[38]. The response can be initiated by excitation of intrinsic neurons and does not appear dependent on the rostroventrolateral medulla. The pathways and transmitters that mediate the effect are unknown. The neuroprotection afforded by this network is long lasting, persisting for almost 2 weeks, and is associated with reduced excitability of cortical neurons and reduced immunoreactivity of cerebral microvessels. This mode of neuroprotection, moreover, is not restricted to focal ischemia, as it has been demonstrated that it also protects the brain against global ischemia and excitotoxic cell death.

That the brain may have neuronal systems dedicated to protect itself from injury, at first appearing to be a novel concept, is, on reflection, not surprising since the brain is not injured in naturalistic behaviors characterized by very low levels of regional CBF, like diving and hibernation. The last phenomenon is one example of manifestation of a tolerance strategy, which is now revealed in all mammals in difficult situations. The metabolic basis of this strategy is the decrease of catabolic energetic expenditure, oxygen, and endogenous mono- and polymers consumption. The ischemic tolerance increases, considerably and steadily, extrinsic and intrinsic factors in the brain. This very perspective approach is reliable for greater results. An important index of ischemic tolerance is, for example, development of hypothermia.

As it is generally accepted that the diving reflex and ischemic tolerance appear to involve, at least partially, similar physiological mechanisms, the existence of such endogenous neuroprotective strategies may extend the actual known clinical appearance of the TCR and include the prevention of other potential brain injury states as well[32,44,45]. This may be in line with the suggestion that the TCR is a physiological, but not a pathophysiological, entity.

### DETAILED EXPERIMENTAL PLAN

#### Animal Model

The present animal model is well established and has been used in different leading universities all over the world during the last decades. The animal model presented here is only adapted to this already established model; its main characteristics have been published recently[28,35].

#### Animal Preparation

The animals were treated according to the recommendations of the National Society for Medical Research ("Principals of Laboratory Animal Care") and the Guidelines of the National Academy of Science ("Guide for the Care and Use of Laboratory Animals", NIH publication No.86-23, revised 1985).

Male cats were anesthetized with ketamine hydrochloride (25 mg/kg) by intramuscular injection. The left femoral vein and artery were catheterized to measure mean arterial blood pressure (MABP), to obtain samples for determining arterial blood gas levels, hemoglobin, and plasma glucose concentration, as well as to administer drugs as necessary and as previously described[35]. The animals were tracheostomized, immobilized with 0.2-mg/kg pancuronium bromide, and artificially ventilated. General anesthesia was maintained by 0.6–1.2% halothane in a 70% nitrous oxide-30% oxygen gas mixture. An intravenous infusion of 2 ml/kg/h Ringer's solution containing 5 mg/kg/h gallamine triethiodide for muscle relaxation was maintained throughout the experiments. Physiological variables were kept in the normal range known for awake cats[48]. Deep body temperature was kept at 37°C using a heating blanket feedback-controlled by a rectal temperature probe.

One hair of the cat's walrus moustache was fixed, so that one may pull on it repetitively. The anesthetized cat is placed in a prone position and fitted with a custom-designed head holder inside the magnet, as previously described[32]. Images were acquired with the use of a 20-cm volume coil as the transmitter coil and a separate 2-cm surface coil for signal detection.

### Experimental Protocol

Routine monitoring of physiological parameters included measurements of heart rate and MABP. In addition, end-tidal concentration of $CO_2$ ($P_{ET}CO_2$; range: 3.5–4.5 kPa) and halothane ($P_{ET}$HAL; range: 0.8–1.2 kPa) were monitored by use of an infrared gas analyzer (Datex, Helsinki, Finland). All parameters were continuously recorded using a PC-based data acquisition system (Dasy Lab, National Instruments, U.S.).

Imaging studies of DWI and T2WI (T2-weighted imaging), as well as FAIR (flow-sensitive alternating inversion recovery) and DSC (dynamic susceptibility contrast), were performed before animal preparation. After animal preparation, the same imaging studies were obtained just before, during, and 24 h after occurrence of the TCR (see Fig. 1).

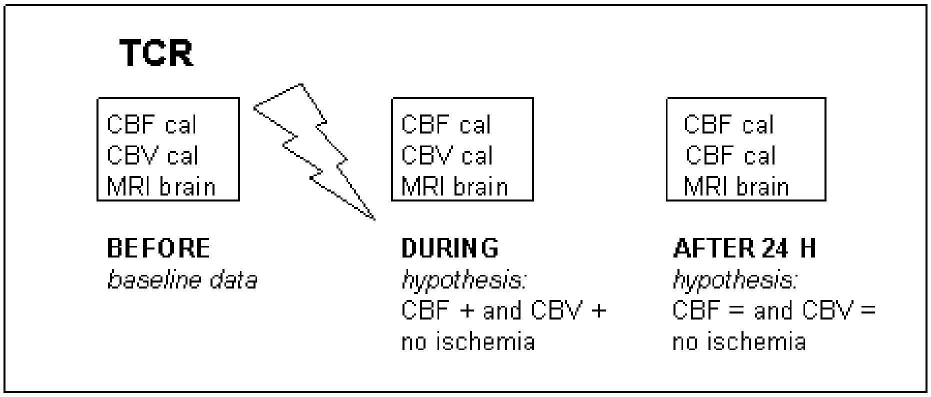

**FIGURE 1**. Plan of the study protocol. CBF: cerebral blood flow; CBV: cerebral blood volume; MRI: magnetic resonance imaging; cal: calculated; +: increased to baseline data; =: unchanged to baseline data.

Each experiment was terminated by perfusion fixation with 4% paraformaldehyde solution under maintenance of general anesthesia. In each cat, the brain was carefully removed from the skull and embedded in paraffin.

## SPECIFIC METHODS

### Evaluation of Ischemic Lesions by DWI and T2WI

Multislice axial T2WI and DWI was scanned with a field of view of 4 cm, a slice thickness of 2 mm, and a matrix size of 256 x 128. The offset was set corresponding to the section –0.3 mm from the bregma, as illustrated in a cat brain atlas[47]. The T2WI was acquired with a repetition time (TR) of 4 sec and six echo times (TE) (20, 40, 60, 80, 100, and 120 msec) to generate T2 maps. The DWI was acquired with the use of a diffusion-weighted stimulated echo sequence with a TR of 2 sec, TE of 43.5 msec, mixing time (TM) of 84.8 msec, Δ of 100 msec, and Δ of 10 msec. Four b values (0, 500, 1000, and 1400 s/mm$^2$) were used to calculate the apparent diffusion coefficient (ADC) along each of the three orthogonal diffusion gradient axes (*x*, *y*, and *z*)[49].

### Assessment of Relative CBF and Relative CBV

Relative CBF was evaluated with FAIR techniques[42]. The FAIR experiment was performed with the use of two inversion recovery–fast spin-echo (IR-FSE) sequences with and without a slice-selective gradient during an inversion pulse. Two slice and nonslice IR-FSE images were collected with a TR of 3 sec, TE of 20 msec, effective TE of 50 msec, echo train length of 4, slice thickness of 2 mm, field of view of 4 cm, inversion time of 1.5 sec, and matrix size of 256 x 128. A slab thickness of 5 mm was inverted for the slice IR-FSE images.

Relative CBV was studied with DSC MRI[43,50]. A series of 40 gradient-echo, 2-mm-thick, transverse, single-slice images with a TR of 30 msec, TE of 10 msec, pulse angle of 15°, and matrix size of 256 x 64 was acquired. The bolus of the susceptibility contrast agent gadolinium diethylenetriamine pentaacetic acid was injected intravenously 10 sec after the start of image acquisition.

## DISCUSSION

The presented animal model is the first that extensively investigates the TCR as defined by our strict criteria (see Table 1). However, Ichinohe et al.[37] presented an earlier rabbit model, in which the hemodynamic changes ameliorated including rCBF reduction during the TCR as compared to a control group, but their stimulation of afferent reflex arc is not a classical TCR, as in our model. Others like White et al.[7] and partly Leonard et al.[56] have only measured the blood flow in the central ear artery and have estimated a CBF that seems not to be a valuable model for exact CBF examination. All previous animal models have not measured a real CBF, so that these results are not really useable. For this reason, our model is the first animal TCR model that allows us also to draw comparisons to humans. In addition, our animal model sheds light on the important aspect of the physiology of CBF, a topic that is under-represented in basic neuroscience research. The relative simplicity of the model allows a wide use.

**TABLE 1**
**Summary of Previous Animal Studies Investigating Cerebral Hemodynamic Changes after Initation of the TCR**

| Study | Animals | Anesthetics | "CBF" Measurement | Results |
|---|---|---|---|---|
| White et al.[7] | Rabbits | Pentobarbiton | Central ear artery | Reduced flow |
| Ichinohe et al.[37] | Rabbits | Halothan | Common carotid artery | Reduced flow |
| Leonard et al.[56] | Rabbits | Pentobarbiton | Central ear artery | Reduced flow |

The initiation of the TCR is followed by a decrease of heart rate and MABP of 20% or more[3,25,26,27]. There exists only sparse knowledge about its influences on CBF and/or cerebral metabolism. The existing papers only measured local cortical changes, but no regional changes of white and gray matter. Until now, there exists no clinical or experimental work that has examined this question serially and in a (semi-)quantitative manner. Corresponding to similar data to cold reflex[13], one may assume an initial, but small, decrease of CBF that may be constant in the follow-up[4], suggesting that active venoconstriction contributes to CBV changes. We are planning to examine the CBF and the cerebral metabolism by cerebral MRI serially to clear the question of what happens to CBF and cerebral metabolism during and after the initiation of the TCR in relation to the "basal state" before initiation the TCR. The TCR will be initiated by specific stimulation of the cat's walrus moustache (manuscript in preparation). To achieve our goal, DWI and T2WI were used to demonstrate the extent of the brain from acute to chronic stages, while PWI was used to measure rCBF and CBV over time. The changes of CBF and CBV over time were then correlated with those in heart rate and MABP. Answering this question is of importance in order to better understand the TCR itself and to develop prophylactic procedures.

The finding that different (sensory) stimuli induce similar effects that habituate markedly on repetition is typical for brainstem reflexes, especially for other trigeminal reflexes, such as the trigeminalfacial reflexes[51]. For the TCR, one may assume, therefore, that interindividual differences on (para)sympathetic and blood pressure effects may be part of interindividual differences in behavior responses to stress (unpublished recent experimental results of our group). However, nothing is known of a "habituation" effect of the TCR. One may assume that the result after initiation of the TCR by stimulation of the cat's walrus moustache may be uniform. As we do not plan to perform electrophysiological testing initially, any indirect answers are not expected. One may assume that the CBF may demonstrate "habituation", and that the cardiac (heart rate) and vascular (MABP) blood flow may have no "habituation". This observation would underline the hypothesis that the cerebral answer may be related to ischemic preconditioning and that there exists no real habituation of the reflex. We plan to provoke five repeated inductions of the TCR by stimulation of the cat's walrus moustache in order to compare their effect on heart rate, MABP, and CBF with the data of a single induction as measured by cerebral MRI. Answering these questions would give important insights into the relationship between the reflex and the (para)sympathetic system and the consequences of its derangement. Depending on the experimental results obtained, one has to discuss the secondary use of electrophysiological methods that are not primarily of interest in this context, as we are only interested in the changes of CBF and the cerebral metabolism in relation to the decrease of heart rate and MABP.

Induction of ischemic tolerance in humans is associated with the number and severeness of the initial stimulus, as we recently demonstrated to transient ischemic attacks in stroke by MRI[36,52,53]. We plan to investigate if there is any correlation of this phenomenon to the TCR by serial MRI. In this context, it may be interesting to examine if an ischemic lesion may be induced by the TCR, as this would underline the hypothesis that the TCR is correlated with ischemic tolerance of the brain. This is still only an hypothesis, not a fact, so proofs are wanted. To solve this problem, further extensive research is necessary.

The physiological function, if there is any, of the TCR remains yet unclear[4]. The similarity of the TCR with the diving reflex[3] lets us draw parallels to the induction of the ischemic tolerance in animals for the TCR that is relatively certain in the case of the diving reflex[46]. This question can be best answered by serial MRI. The answer to this question is essential as it will open the window to potential therapeutic options of different neurological disorders[34]. In this context, we would only like to mention that stimulation of the baro-reflex is currently used successfully to treat arterial hypertension[54,55].

After answering these questions, the influence of the TCR on CBF and cerebral metabolism should be better known and may be the basis for further studies, experimentally or clinically. One may assume that the outcome of skull base operations may be substantially improved by this better knowledge of the behavior of the TCR[10]. For us, as neurosurgeons, this new knowledge of the side effects may be therapeutically used for other neurological disorders, such as stroke[4].

### CONCLUSION

We present for the first time an animal model that investigates the TCR. This is important as the TCR has now gained great clinical interest in skull base surgery, but there is a lack of understanding of the underlying mechanisms. For this reason, the present animal model may help us to gain further insights into the physiology of the TCR and may promote a better understanding of the CBF under stress conditions.
